# Effects of Synbiotics among Constipated Adults in Serdang, Selangor, Malaysia—A Randomised, Double-Blind, Placebo-Controlled Trial

**DOI:** 10.3390/nu10070824

**Published:** 2018-06-26

**Authors:** Ying Jye Lim, Rosita Jamaluddin, Abu Saad Hazizi, Jin Yu Chieng

**Affiliations:** 1 Department of Nutrition and Dietetics, Faculty of Medicine and Health Sciences, Universiti Putra Malaysia, 43400 UPM Serdang, Selangor, Malaysia; yingjye806@gmail.com (Y.J.L.); hazizi@upm.edu.my (A.S.H.); 2 Gastroenterology and Hepatology Unit, Department of Medicine, Faculty of Medicine and Health Sciences, Universiti Putra Malaysia, 43400 UPM Serdang, Selangor, Malaysia; cjy511@yahoo.com

**Keywords:** functional constipation, Rome III criteria, Bristol Stool Form Scale, defecation frequency, synbiotics, probiotics, prebiotics, *Lactobacillus plantarum* LP01, *Bifidobacterium lactis* BB12, randomised controlled trial (RCT)

## Abstract

Synbiotics approach complementarily and synergistically toward the balance of gastrointestinal microbiota and improvement in bowel functions. A randomised, double-blind, placebo-controlled study was conducted to examine the effects of a synbiotics supplement among constipated adults. A total of 85 constipated adults, diagnosed by Rome III criteria for functional constipation were randomised to receive either synbiotics (*n* = 43) or placebo (*n* = 42) once daily (2.5 g) in the morning for 12 weeks. Eight times of follow-up was conducted every fortnightly with treatment response based on a questionnaire that included a record of evacuation (stool frequency, stool type according to Bristol Stool Form Scale), Patients Assessment on Constipation Symptoms (PAC-SYM), and Patients Assessment on Constipation Quality of Life (PAC-QOL). There were no significant differences in stool evacuation, but defecation frequency and stool type in treatment group were improved tremendously than in placebo group. While the treatment group was reported to have higher reduction in severity of functional constipation symptoms, the differences were not statistically significant. Dietary supplementation of synbiotics in this study suggested that the combination of probiotics and prebiotics improved the functional constipation symptoms and quality of life although not significant. This was due to the high placebo effect which synbiotics failed to demonstrate benefit over the controls.

## 1. Introduction

Functional constipation (FC) is characterised by infrequent defecation of fewer than three defecations in a week and difficulty in defecation with hard or lumpy stool [1,2]. It is a common functional gastrointestinal disorder (FGID), which affects the people world-widely. Digestive system disease is the top ten principal causes of morbidity and mortality among Malaysian whereby colorectal is the most frequent incident reported [3,4,5,6,7,8]. Functional constipation is affected by diet [9,10], lifestyle [11,12,13], psychological [12,14,15], and socio-demography factors [12,16]. The incidences of FC also negatively impact the quality of life of the respective individuals.

A probiotic is defined as “live microorganism which when administered in adequate amounts confers a health benefit on the host” [17]. International Scientific Association for Probiotics and Prebiotics (ISAPP) describes probiotics as type of food supplements for human [18]. Therefore, probiotics have been regarded with their health enhancing properties as functional food with natural live microbes that are beneficial toward human gastrointestinal (GI) tract [19,20]. In this study, *Bifidobacterium lactis* BB12 and *Lactobacillus plantarum* LP01 are the probiotics strains that used. BB12 is Generally Recognized as Safe (GRAS) by the Food and Drug Administration (FDA) in the United States [21]. It is one of the commonest probiotic strain used for supplement and food application [22], due to its ability to survive until consumption, high acidic and bile tolerance [23], inhibition of pathogens and ability to colonise [22,24,25,26], improvement in immunity against GI infections [27], and no reported adverse effects on taste, appearance or mouthfeel [22]. Importantly, BB12 adhesiveness in intestinal mucin [28,29] has made it an excellent strain to enhance GI health, which was revealed from the internal laboratories tests of Hansen [22]. Overall, BB12 were clinically tested to improve bowel functions. BB12 was revealed to normalise bowel movement [30], improve defecation frequency [31,32,33,34], and increase fecal count of bacteria BB12 [35].

As for *Lactobacillus plantarum* LP01, it has been clinically proven to improve bowel function [36,37], possess immunomodulation effects [38], prophylactic properties [39], and inhibition activity of *Escherichia coli* [40] indicated that LP01 can antagonize potential intestinal pathogens. Trials conducted using LP01 showed that significant improvement in defecation frequency, constipation symptoms (stool consistency, ease of defecation, sensation of incomplete emptying, and sensation of itching, burning, or pain during or after defecation) [36], fecal counts of probiotic strain [37] when compared with placebo group. Furthermore, the ability of LP01 to produce short chain fatty acids (SCFA) has ensured the ability of the respective strain to maintain GI microflora and reduced the enzymatic activities as well as generation of carcinogens [41]. Based on the report of European Food Safety Authority (EFSA), LP01 has the ability to produce β-galactosidase in high amount that helped in lactose digestion and fermentation of prebiotics which then improved in intestinal mobility [42]. Furthermore, LP01 is also a suitable probiotic strain in forming synbiotics products [39]. Thus, with the combination of other specific probiotics strains and prebiotic, LP01 helps to reduce GI discomfort resulted from increased transit time as well as relieve abdominal discomfort and pain [42].

Prebiotics, the food source for probiotics, are defined as “non-digestible food ingredients that beneficially affect the host by selectively stimulate the growth and/or activity of one or a limited number of bacteria in the colon, thus improving host health” [43]. Ingestion of prebiotics could promote the growth of good bacteria and enhance the microbiota in the intestinal tract [43], as well as supply constipation alleviation effect [44,45] towards individuals with bowel irregularity without causing any distress in their GI [45]. Inulin-oligofructose was the prebiotic used in this study. It was found to be the specific substrates for the growth of bifidobacteria [46,47], thus making it an ingredient with bifidogenic functional properties. Inulin-oligofructose was well studied in their utilization by human colonic butyrate-producing bacteria. Thus, the growth of GI bacteria and gut health can be enhanced [48].

In combination of probiotics and prebiotics, synbiotics are referred as food ingredients or dietary supplements which can form synergism within the GI tract [49]. Hence, Food and Agriculture Organisation (FAO) emphasized the use of the term “synbiotic” is allowed if there is synergistic health effects [50]. Modification of micro-environment by synbiotics can improve the condition of functional constipation [51] inclusive of increase in defecation frequency, stool consistency improvement, shorten of transit time and other constipation-related symptoms [52]. In the present study, a prospective, randomised, double-blind, placebo-controlled study was conducted to examine the effects of synbiotics supplement (combination of BB12, LP01, and inulin-oligofructose) on functional constipation symptoms among the respective individuals.

## 2. Materials and Methods

### 2.1. Study Design

This study was a 16-week randomised controlled trial (RCT), with double-blinding procedure and parallel groups applied. Screening for functional constipated individuals was conducted in Serdang, Selangor, Malaysia. Screening and participants recruitment were carried out simultaneously. Potential participants were identified through a cross-sectional survey based on Rome III diagnostic criteria and were further contacted to attend a clinical review session with gastroenterologist, followed by a blood test. Only those who met the strict eligibility criteria were invited to participate in the study.

Written informed consents were obtained and the recruited participants were randomly allocated to either group A or B, which was later revealed as synbiotics treatment and placebo control group, respectively. The sealed envelope method was used for randomisation process. A third-party controller was assigned to hold the code. Both researchers and participants were blinded to the allocation groups and revealed only after the analysis was completed.

After a 2-week baseline, participants received synbiotics or placebo once daily for 12 weeks. Study supplements were delivered to the participants once every fortnight. Another two weeks of post-intervention was conducted to obtain the final assessment on the participants. Participants were advised to maintain their normal diet and lifestyle, but to avoid other probiotics and synbiotics products, dietary fiber supplements, and laxatives throughout the study.

### 2.2. Ethical Considerations

The study was carried out in accordance to Malaysian Guidelines for Good Clinical Practice (GCP), the Declaration of Helsinki. Ethical consideration and approvals to conduct the study were obtained from the Ethic Committee for Research Involving Human Subject (JKEUPM) from Universiti Putra Malaysia (Reference Number FPSK(FR14)CT002) and the Ministry of Health (MOH) Medical Research Ethics Committee (MREC) (National Medical Research Registry (NMRR) Research ID NMRR-14-1612-19895 (IIR)).

### 2.3. Safety Considerations

In the absence of bowel movement for four consecutive days or more, syrup lactulose was assigned as rescue therapy for the participants as a safety consideration.

### 2.4. Study Agents

The supplement for the treatment group is synbiotics whereas placebo for control group. Both synbiotics and placebo were prepared in 2.5 g sachet with identical physical form and odour. Synbiotics supplement consisted of 10 billion colony forming unit (CFU) *Lactobacillus plantarum* LP01, *Bifidobacterium lactis* BB12, and inulin-oligofructose. As for the placebo, it was made up of maltodextrin without any active ingredients and prepared by the same manufacturer.

### 2.5. Eligibility

Participants were eligible to enroll in the study if they fulfilled the rigorous inclusion and exclusion criteria:

Inclusion criteria: Malaysian of either gender; aged 18 to 65 years old; body mass index (BMI) of 16.0 to 29.9 kg/m^2^; positively diagnosed with functional constipation with Rome III-defined constipation module by the gastroenterologist.

Exclusion criteria: Vulnerable groups of pregnant and breastfeeding women; physically or mentally handicapped individuals; diagnosed with organic constipation (constipation associated with any neoplastic diseases, neuropathy or mechanical obstruction); diagnosed with cardiovascular diseases, diabetes mellitus, cancer, neurological disease or other serious illnesses or severe medical complications; faced alarm features indicative of colorectal cancer, metabolic disease and a history of gastrointestinal surgery. To avoid the likelihood of co-intervention bias, the following respondents were excluded: on gastrointestinal medications; consumed probiotics or synbiotics products more than once a week in two weeks preceding screening or during intervention period; regularly used laxatives (more than once per week), used anticholinergic, anti-diarrheal, antibiotics or laxative in two weeks preceding screening or at any point during intervention.

### 2.6. Efficacy Measurements

The primary outcome measure of the present study was the improvement in weekly defecation frequency. The secondary outcome measures were improvement in stool type based on Bristol Stool Form (BSF) Scale and Patients Assessment on Constipation Symptoms (PAC-SYM) scores. In addition, quality of life among the individuals with constipation was measured through Patients Assessment of Constipation Quality of Life (PAC-QOL).

BSF Scale is a pictorial scale used to characterise human stools to seven types of classification which represents different bowel transit time. Type 1 stool indicates very slow transit time with the longest time spent in the colon; Type 7 shows the least time spent in the colon with the fastest bowel transit time which is entirely liquid; whereas Type 4 is considered as the most ideal stool type that resembles individuals with daily defecation [53].

PAC-SYM scores were computed using a questionnaire with three domains which included abdominal, rectal, and stool symptoms. It was measured using a 5-point Likert scale with lower score obtained indicates less symptom severity. PAC-QOL scores were calculated based on a four domains questionnaire with 5-point Likert scale, to identify the changes in quality of life among the constipated individuals throughout the study. It included physical discomfort, psychological discomfort, worries and concerns, and satisfaction towards bowel movement. Lower scores indicate better quality of life among the individuals.

### 2.7. Sample Size

Sample size was computed based on primary end-point measure of defecation frequency. The sample size required in this study was determined by referred to the randomised controlled trial conducted by Fateh et al. that applied four weeks synbiotics treatment to improve functional constipation symptoms [54]. Considered the standard deviation of difference after intervention was 0.8 and mean difference between treatments of 0.6 defecation frequency per week, a total of 30 participants was required for each group with 5% significance level at 80% of power of the test was set. To include a 20% of drop-out rate, a minimum of 76 participants was required to ensure the final data obtained to be generalised to the population.

### 2.8. Statistical Analyses

The result was analysed based on the intent-to-treat (ITT) principle. All statistical analyses were conducted using SPSS version 22.0 for Windows (SPSS Inc., Chicago, IL, USA). For descriptive data of sociodemographic characteristics, the mean and standard deviation were presented for continuous data whereas frequency distribution with percentiles, *n* (%) for categorical data. Cross-tabulation was used to determine the association between two categorical variables. Chi-square (χ2) test was performed with the condition of expected count less than 5% is less than 20% and minimum expected count more than two, otherwise Fisher’s Exact test was used [55]. Spearman rank order correlation analysis was utilised to determine the relationship between two non-normally distribution of continuous data.

A mixed design ANOVA was conducted using a General Linear Model (GLM) repeated measures test to identify the effects of supplementation on functional constipation symptoms. The GLM repeated measures ANOVA was conducted with the within-subjects factor being time/assessments at two levels and between-subjects factor being groups at two levels (synbiotics supplement versus placebo control). All statistical tests applied with a difference considered to be significant if *p* < 0.05.

## 3. Results

### 3.1. Participants’ Flow

**Figure 1:**
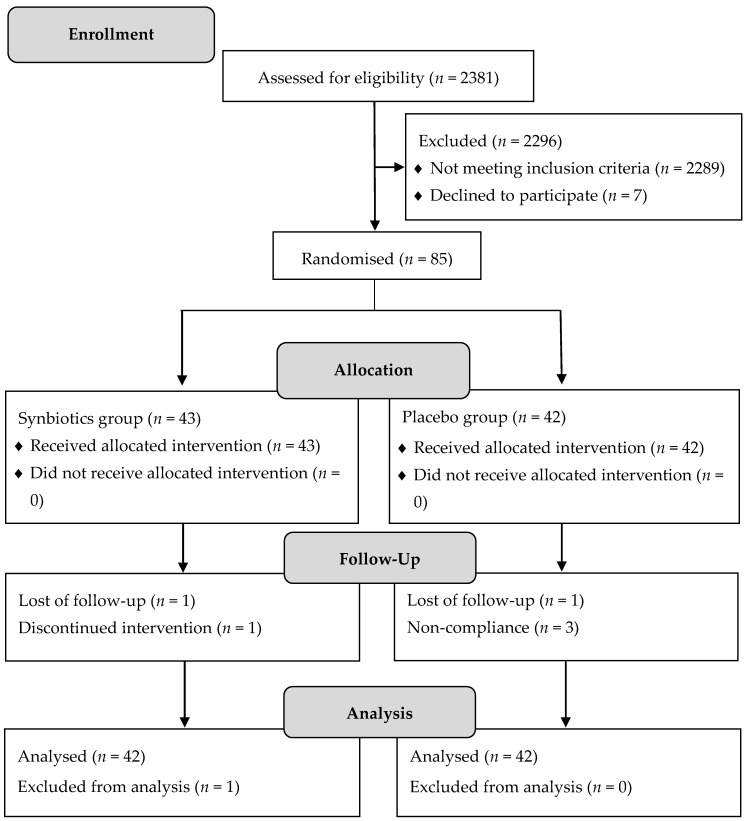
Consolidate standards of reporting trials (CONSORT) flow diagram of participants.

A total of 2381 respondents were screened to identify the eligible participants in the intervention study. Out of 2296 respondents, 2289 respondents did not meet the rigorous eligibility criteria and seven of them declined to participate. Finally, written informed consents were obtained from 85 participants and they were randomised in this intervention study. There were 43 participants assigned to synbiotics treatment group and 42 participants to placebo control group. Figure 1 shows the CONSORT flow diagram that represents the participants’ flow through this trial.

### 3.2. Baseline Characteristics

Of 85 participants recruited, majority of them were women (85.9%) as compared to men (14.1%). The mean ages were 29.5 and 27.5 years for synbiotics treatment and placebo groups respectively. At baseline, participants in two groups were well-matched in term of the socio-demographic characteristics and anthropometric data (Table 1), as well as functional constipation symptoms which included defecation frequency, stool type, PAC-SYM scores, and quality of life (Table 2). There were no significant differences between participants’ baseline characteristics between synbiotics and placebo group at the commencement of the study.

### 3.3. Functional Constipation Symptoms

**Figure 2:**
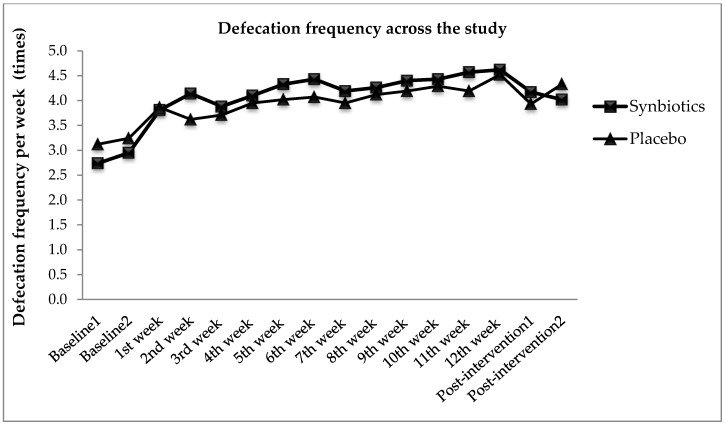
Defecation frequency from baseline, intervention, and post-intervention.

Functional constipation symptoms measured in this study included defecation frequency, stool type, and PAC-SYM scores. Figure 2 illustrates the effects of synbiotics and placebo on defecation frequency from baseline, intervention to post-intervention period. At baseline, the mean defecation frequency for synbiotics and placebo group were 2.7 ± 1.1 times and 3.1 ± 1.0 times per week respectively. The defecation frequency increased during intervention period and reduced at post-intervention period for both groups. From the observation, even though significant improvement in main effect of time [*F*(8.83, 723.83) = 15.53, *p* < 0.001, η*_p_*^2^ = 0.16] on defecation frequency from the result of mixed between-within subjects ANOVA was found, however, there was no significant difference reported when comparing both synbiotics and placebo groups (*p* > 0.05). The result indicated a 12-week treatment period of 10 billion CFU per day of synbiotics in functional constipation individuals did not bring about statistical significant improvement in defecation frequency. Thus, there was no significant main effect on types of supplement groups [*F*(1, 82) = 0.201, *p* = 0.65, η_p_^2^ = 0.003] on defecation frequency, with synbiotics group (mean = 4.07) and placebo group (mean = 3.95) performing similarly overall. Although there was a significant increase in defecation frequency in placebo group, the mean frequency was higher in synbiotics group. Synbiotics group experienced 68.6% increases in defecation frequency as compared with baseline; whereas there was only 44.9% in placebo group. In addition, it can be observed from the intercept in Figure 2 that there was an interaction effect between synbiotics and placebo groups. However, it was not significant [*F*(8.83, 723.83) = 1.33, *p* = 0.22, η*_p_*^2^ = 0.02].

**Figure 3:**
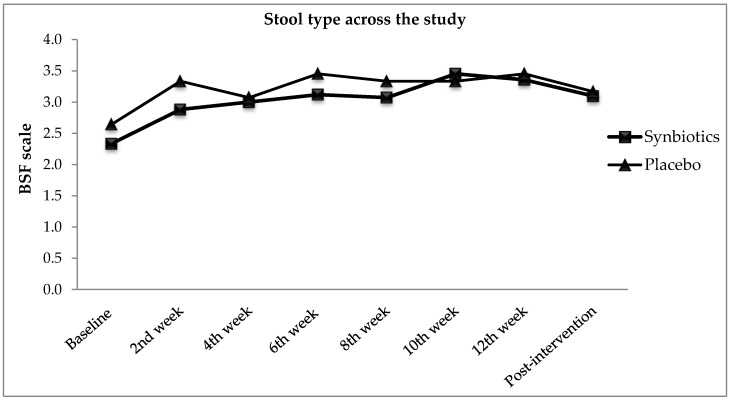
BSF scale from baseline, intervention, and post-intervention.

The effects of types of treatment on stool type were determined through Bristol Stool Form (BSF) Scale and the changes were presented in Figure 3. At baseline, participants in synbiotics group scored 2.3 ± 0.9 for BSF Scale whereas 2.5 ± 1.1 in placebo group. Improvement in stool type was reported throughout the study. Yet, there was no significant difference in stool type found [*F*(1, 82) = 1.07, *p* = 0.305, η*_p_*^2^ = 0.01] when comparing two groups. As both synbiotics (*p* < 0.001) and placebo group (*p* < 0.01) reported significant improvement in stool type as compared with baseline, synbiotics group (47.4%) had higher improvement in stool type than placebo group (31.2%). The effect of follow-up showed the improvement of stool type reported in BSF Scale was significant across the study period [*F*(5.74, 470.79) = 7.84, *p* < 0.0005, η*_p_*^2^ = 0.09] for both groups. The finding hereby indicated the 12-weeks synbiotics supplementation on functional constipation individuals did not significantly affect the BSF scale as compared with placebo group.

**Figure 4:**
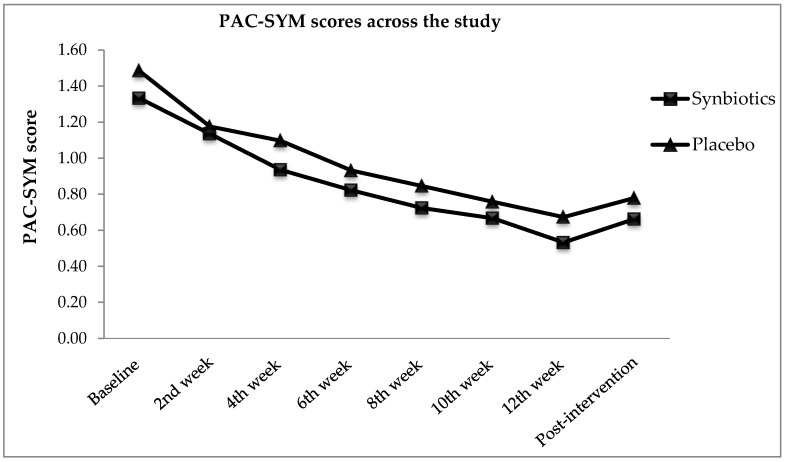
Patients Assessment on Constipation Symptoms (PAC-SYM) scores from baseline, intervention, and post-intervention.

Figure 4 presents the Patients Assessment of Constipation Symptoms (PAC-SYM) scores for both treatment and control group throughout the study. A decreasing trend displayed showing an improvement in PAC-SYM scores. In Figure 4, tremendous decreases were observed in synbiotics and placebo groups. Despite the significant within-subjects effect throughout the intervention [*F*(4.78, 389.25) = 32.07, *p* < 0.0005, η*_p_*^2^ = 0.28], no significant difference was found between the two groups when comparing the PAC-SYM scores reported [*F*(1, 82) = 1.07, *p* = 0.65, η*_p_*^2^ = 0.01]. The synbiotics group reported 49.6% alleviation in PAC-SYM scores, whereas 43.7% reported alleviation in control. The presence of placebo effect in this study was suspected. Synbiotics did not improve the PAC-SYM scores significantly as compared with placebo throughout the intervention period.

### 3.4. Quality of Life

**Figure 5:**
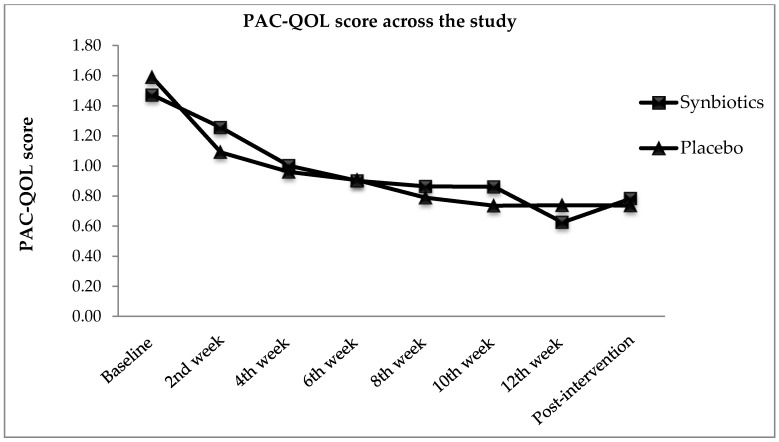
Patients Assessment on Constipation Quality of Life (PAC-QOL) scores from baseline, intervention, and post-intervention.

Changes in quality of life were observed through Patients Assessment on Constipation Quality of Life (PAC-QOL) scores obtained from participants of synbiotics and placebo groups as presented in Figure 5. With lower mean score of PAC-QOL indicated better quality of life, the decreasing trend displayed in Figure 5 showing that participants in both synbiotics and placebo groups experienced improved quality of life. Even though there was significant improvement in quality of life in synbiotics group as compared with baseline (*p* < 0.001), but changes in PAC-QOL scores when comparing two groups was not significant [*F*(1, 82) = 3.51, *p* = 0.065, η*_p_*^2^ = 0.04]. The finding showed that synbiotics do not effectively change the quality of life when compared with placebo. Again, placebo group reported with significant improvement in quality of life too (*p* < 0.001). There were 33.2% improvement of PAC-QOL scores in synbiotics group and 17.0% in placebo group. Overall, it can be observed that the effect of supplementation within group was significant during the trial [*F*(4.51, 369.89) = 13.17, *p* < 0.0005, η*_p_*^2^ = 0.14].

## 4. Discussion

This study was a randomised controlled trial to examine the effects of a combination of probiotics *Lactobacillus plantarum* LP01, *Bifidobacterium lactis* BB12, and prebiotics inulin-oligofructose among functional constipation individuals diagnosed through Rome III criteria. Studies conducted using LP01 have proven with positive effects in improving defecation frequency and constipation symptoms [36], as well as increased fecal count [37]. In addition, BB12 has been well studied in improving gastrointestinal health which included bowel regularity [30,32], defecation frequency [31,33,34], and intestinal microflora [33,34]. As for inulin-oligofructose, it has been proven to increase overall GI score [56] and increase fecal bifidobacteria [57,58].

Prior to the intervention study, a cross-sectional study has been carried out as part of this project to identify the eligible participants. As part of the study, the prevalence of functional constipation among respondents from tertiary education institution in Malaysia was revealed as 16.2% [59]. In the present study, a 12-week treatment period with a combination of LP01, BB12, and inulin-oligofructose which formed synergistic effects was found to significantly improve the functional constipation symptoms within the synbiotics group. Nonetheless, there was significant improvement in placebo group too. Yet, there was no significant difference reported when comparing treatment and control groups. Improvement of functional constipation symptoms in synbiotics group could be further elaborated by the reduction of whole gut transit time (WGTT) and intestinal colonic transit time through ingestion of the supplement [60,61,62]. Therefore, synbiotics supplementation could increase the defecation frequency among the individuals. For the improved defecation frequency, the finding of this trial was in line with the previous study conducted whereby both synbiotics and placebo groups were found to improve constipation significantly as compared with baseline but not significant between treatment and control groups [63]. The positive effects within the placebo group might be due to the variation in the subjects’ daily diet, such as consumption of spicy foods which could irritate the bowel [63], changes in physical activity level [11,64], changes in quality of life [65,66], as well as changes in psychological factors [12,15].

Ingestion of synbiotics promote the colonic motility and stimulate the defecation process which then reduce the intestinal transit time and minimise the water reabsorption in the colon. Thus, synbiotics supplementation has illustrated changes in stool type among the functional constipation participants throughout the intervention. However, there was no significant difference demonstrated in the present study when comparing the changes in stool type between synbiotics and placebo groups. This finding is again in agreement with the regional study conducted using probiotics fermented milk in constipation [63], which shows no significant difference when comparing treatment and control groups. Yet, another local study using microbial cell preparation in constipation has proven with significant improvement in stool consistency as compared with placebo group [67]. The increased probiotics in GI tract can promote the secretion of water and electrolytes which then soften the stool [68]. Thus, the stool type changed from hard to defecate to easier to glide out through synbiotics supplementation.

Synbiotics execute a synergistic interaction effect and promote the growth of live bacteria that improve constipation symptoms [69] and this was observed with the alleviation in Patients Assessment of Constipation Symptoms (PAC-SYM) scores. Production of short-chain fatty acids from fermentation process has stimulated the bowel movement [70]. Thus, functional constipation symptoms were improved with softer stool, less rectal bleeding or burning, and less painful defecation. Meanwhile, the improvement in functional constipation symptoms in control group was suspected with placebo effect.

Negative impacts of constipation in quality of life were widely reported. Constipation was reported with negative quality of life and social consequences [71,72,73,74]. Throughout the treatment period, participants in synbiotics group were reported with improved functional constipation symptoms as well as quality of life. Surprisingly, participants in the placebo group were reported with improved quality of life, too. Nonetheless, placebo effect was present in the current study. As quality of life is highly correlate with clinical responses such as functional constipation symptoms [65,66], thus it was postulated that improvement in functional constipation symptoms among participants in the placebo group could be counterfeited by the increased quality of life.

However, our study had certain limitations to be pointed out. Treatment dosage at different concentration may provide a significant result. Compliance was assessed through participants’ feedback. Fecal sample was not collected to assess the supplement compliance or to examine the fecal microbiota and short chain fatty acids as resulted from synbiotics supplementation. Otherwise, the presence of probiotics in feces would scientifically disclose on the compliance and the viability of the probiotics which further strengthen the study observation.

## 5. Conclusions

Further randomised, controlled studies are required to affirm whether the synbiotics supplement that contained 10 billion CFU *Lactobacillus plantarum* LP01, *Bifidobacterium lactis* BB12, and inulin-oligofructose is effective in improving functional constipation symptoms and quality of life as compared with placebo. However, the overall findings of the present study seem to support the use of synbiotics supplement to improve functional constipation among the respective individuals rather than laxative which can result in life-threatening complications. Placebo group was revealed with improvement not only in functional constipation, but also quality of life. This was due to the high placebo effect, for which synbiotics failed to demonstrate benefit over the controls.

## Figures and Tables

**Table 1:** Participants’ socio-demography and anthropometric data at baseline.

Characteristics	Synbiotics (*n* = 43)	Placebo (*n* = 42)	*p*-Value
Sex *			
Male	7 (16.3)	5 (11.9)	0.533
Female	36 (83.7)	37 (88.1)	
Age (years) ^φ^	29.5 ± 8.34	27.5 ± 6.5	0.368
Ethnicity *			
Malay	36 (83.7)	38 (90.5)	0.332
Non-Malay	7 (16.3)	4 (9.5)	
Weight (kg) ^φ^	59.4 ± 11.7	58.7 ± 12.6	0.911
Height (m) ^φ^	1.59 ± 0.09	1.59 ± 0.09	0.738
BMI (kg/m^2^) ^φ^	23.6 ± 4.1	23.6 ± 4.1	0.578
Waist circumference (cm) ^φ^	80.0 ± 9.5	80.0 ± 9.5	0.977

BMI: body mass index. * Values are expressed as *n* (%); ^φ^ Values are expressed as mean ± standard deviation.

**Table 2:** Participants’ functional constipation symptoms and quality of life at baseline.

Characteristics	Synbiotics (*n* = 42)	Placebo (*n* = 42)	*p*-Value
Functional constipation symptoms			
Defecation frequency ^φ^			
Baseline week-1	2.7 ± 1.1	3.1 ± 1.0	0.148
Baseline week-2	2.9 ± 1.2	3.2 ± 1.3	0.268
BSF scale ^φ^	2.3 ± 0.9	2.6 ± 1.3	0.352
PAC-SYM score ^φ^	1.40 ± 0.72	17.93 ± 7.76	0.525
Quality of life			
PAC-QOL score ^φ^	1.53 ± 0.67	35.8 ± 16.9	0.736

BSF: Bristol Stool Form; PAC-SYM: Patients Assessment on Constipation Symptoms, and PAC-QOL: Patients Assessment of Constipation Quality of Life. ^φ^ Values are expressed as mean ± standard deviation.
